# An exploration of impaired walking dynamics and fatigue in Multiple Sclerosis

**DOI:** 10.1186/1471-2377-12-161

**Published:** 2012-12-27

**Authors:** Janina M Burschka, Philipp M Keune, Uwe Menge, Ulrich Hofstadt-van Oy, Patrick Oschmann, Olaf Hoos

**Affiliations:** 1 Institute of Sports Science, University of Bayreuth, 95440, Bayreuth, Germany; 2 Klinikum Bayreuth GmbH, Betriebsstätte Hohe Warte, Department of Neurology, Hohe Warte 8, 95445, Bayreuth, Germany; 3 Department Medicine, Training and Health, Institute of Sports Science, Philipps-University Marburg, Marburg, Germany

**Keywords:** Multiple sclerosis, Ambulation, Walking, 6 minute walk, 6MW, 12 minute walk, 12MW, Linear trend, U-shape

## Abstract

**Background:**

Physical disability in multiple sclerosis (MS) is frequently characterized by impaired ambulation. Although walking tests have been successfully employed to assess walking ability in MS patients, data analytic procedures have predominantly relied on result-oriented parameters (e.g. total distance covered during a given amount of time), whereas process-oriented, dynamic walking patterns have mostly been ignored. This is striking, since healthy individuals have been observed to display a stereotypical U-shaped pattern of walking speed during timed walking, characterized by relatively high speed during the initial phase, subsequent slowing and final acceleration. Objective of the current study was to test the utility of the 6 min Walk (6MW) and the 12 min Walk (12MW) for revealing putatively abnormal temporal dynamic features of walking in MS.

**Methods:**

A group of 37 MS patients was divided into subgroups with regard to their level of disability analyzed with the Expanded Disability Status Scale (EDSS; Mild MS Group, n = 20, EDSS 0 – 3.5; Moderate MS Group, n = 17, EDSS 4 – 5). Subsequently, both groups were compared to age-matched healthy controls (n = 25) on both tests with regard to result-oriented characteristics (mean walking speed), as well as dynamic features (mean decline in walking speed, degree of observed U-shape).

**Results:**

Both MS groups showed a significantly lower mean walking speed than healthy controls, independent of test duration. Compared to controls, the Moderate MS Group also slowed down more rapidly throughout both tests. The same pronounced decline in walking speed was observed for the Mild MS Group in case of the 12MW. Additionally, for both MS groups an attenuated U-shaped velocity pattern was observed relative to controls in the 6MW. Patients' subjective fatigue scores were more strongly correlated with the decline in walking speed than with the common parameter of mean walking speed in the 6MW.

**Conclusions:**

MS patients display abnormal dynamics in their walking patterns. A pronounced linear decline in walking speed can be identified with the 12MW even in MS patients with seemingly mild disability. Similarly, the 6MW can be used to assess an abnormal walking profile. Particularly the linear decline in walking speed on this test shows a more robust association with subjective fatigue than mean walking speed. Dynamic walking parameters may hence represent valuable clinical features, serving as surrogate measures of motor fatigue. Future studies are needed to verify their prognostic value.

## Background

A wide range of tests is available to measure walking performance in patients suffering from Multiple Sclerosis (MS; see Kieseier et al. [1] and Bethoux et al. [2] for recent reviews). Among the most widely used tests are the timed 25 Foot Walk, which measures the time a patient requires to cover a distance of 25 feet at maximum speed, and the 6 min Walk (6MW), which measures the total distance a patient is able to cover walking as fast as possible for six minutes. Particularly the 6MW is commonly administered and has been shown to display external validity, as performance on this test is associated with balance confidence and stair-climbing ability [3].

The application of the 6MW has consistently revealed that MS patients may display considerable walking deficits. Further, abnormal physiologic responses during walking performance were reported. Savci et al. [4] for example observed a significantly lower mean walking speed during the 6MW in MS patients (n = 30, score on the expanded disability status scale [5] EDSS = 1.5 – 6.0) compared to controls (n = 30). The same pattern was observed by Chetta et al. [6], who additionally reported reduced cardiorespiratory fitness, i.e. decreased oxygen pulse and inefficient ventilation, as reflected in an impaired breathing pattern in MS patients (n = 11, EDSS = 1 – 3.5) compared to controls (n = 10). These differences were observed despite normal cardiopulmonary function at rest.

According to Motl et al. [7], even mildly disabled MS patients may differ significantly from healthy controls in their mean walking speed on the 6MW. Besides differences in speed, a differential pattern of oxygen consumption between the first and the second half of the test was observed [8]. The authors concluded that different metabolic systems may be relevant for performance during the first and the second half of the 6MW.

Findings reported by Motl et al. [8] are particularly interesting since parameters which are commonly derived from walking tests (e.g. mean walking speed or total distance covered) are not suitable to examine walking characteristics which may vary during the test. In very few studies, an attempt was made to examine dynamic walking features, such as the progression of walking speed throughout the test duration. In an early study, Schwid et al. [9] reported a significantly lower median walking speed in MS patients measured over a distance of 500 meters. MS patients however did not only walk slower, but also showed a considerable decrease in walking speed towards the end of the test, whereas controls accelerated. Goldman et al. [10] examined velocity profiles of MS patients during the 6MW. Results revealed that MS patients differed from healthy controls in both, the mean walking speed as well as the course of walking speed across the six-minute time span. In case of the control group, a U-shaped velocity profile was observed, with relatively high walking speed at the beginning of the walk, subsequent slowing and acceleration towards the end of the test. In contrast, in MS patients the acceleration toward the end of the test was attenuated [10]. The U-shaped pacing profile has previously been described as a common phenomenon in healthy participants, warranted that the duration of the performed activity is sufficiently long [11]. Hence, the examination of dynamic characteristics, such as the change in walking speed at different stages of the test may provide further, clinically relevant information.

Findings on dynamic walking patterns are also of relevance for the ongoing discourse, whether walking tests could be shortened to provide economically optimized assessment tools for ambulation in MS. Gijbels et al. [12,13] have suggested that an abbreviated version of 2 min duration may suffice to provide an estimate of walking ability. In their study, the authors reported a decline in walking speed during the first three minutes and constant pacing during the consecutive three minutes of the 6MW in a sample of 40 MS patients (EDSS 1.5 – 6.5) [12]. Moreover a separately conducted 2MW showed the same decline in mean walking speed per minute. Nevertheless, findings by Schwid et al. [9], Goldman et al. [10] as well as Motl et al. [8] are not necessarily consistent with this suggestion and imply that shortening of the 6MW might result in the loss of potentially important information of clinical relevance.

In this context, a further exploration of dynamic walking features in MS seems warranted. Even though the observation of a specifically impaired walking profile may be promising for future clinical applications, so far only Goldman et al. [10] have provided information on walking dynamics. The purpose of the current study was to address this issue and to examine the walking dynamics of MS patients more rigorously. In particular, the intention was to replicate results by Goldman et al. [10] and to apply further data analyses for a comparison of walking profiles of MS patients and healthy participants. Based on the suggestion that the duration of walking needs to be sufficiently long to yield a U-shaped pacing profile, in the current study, we examined whether the 6MW, as well as a 12 min Walk (12MW) would reveal distinct dynamics in walking behaviour of MS patients relative to healthy controls. We further collected self-report data with regard to somatic fatigue, in order to verify whether putatively abnormal walking dynamics were related to patient's subjective constraints.

## Methods

A group of 37 MS patients was recruited from the Department of Neurology, Klinikum Bayreuth GmbH, Germany. The comparison group consisted of 25 age-matched healthy participants. Details regarding demographics and clinical characteristics of the sample are displayed in Table 1. All participants provided written informed consent prior to study entry and the study was approved by the ethics committee of the Bavarian Medical Association, Germany. Information about clinical characteristics was extracted from patients’ files held by the Department of Neurology. Walking tests (6MW, 12MW) were administered by a single, highly trained clinical examiner during one or two patient visits. Patients completed the two walking tests in randomized order, with at least 3 hours between the assessments. The administration of the 6MW followed standard protocol [14], with modifications suggested by Goldman et al. [10]. For six minutes, participants walked back and forth a distance of 20m, which was divided into 1m segments marked on a wall. Distance covered during each minute was assessed visually, based on the marked segments, by an examiner equipped with a stop-watch. The examiner was seated ten meters away from the midpoint of the walking distance and recorded the distance covered during each minute. This setting was chosen over other methods, such as accompanying the patient with a precise wheel, in order to minimize a putatively confounding influence of the setting on the participants’ walking behaviour. The 12MW followed the same procedure, with the only difference that the walking duration was twice as long. Additionally, the Wuerzburger Fatigue Inventory for Multiple Sclerosis (WEIMuS) was implemented prior to the first test as a measure of subjective somatic fatigue [15].

**Table 1:** Demographics, clinical information and health behavior

	**MS Patients (n = 37)**		**Controls (n = 25)**		**statistic**	**p-value**
**Demographics**						
Age M(SD)	39.7	(12.8)	38.4	(11.9)	0.40^a^	0.69
Female sex, n (%)	28	(75.6)	18	(72.0)	0.11^b^	0.75
**Health Behavior**						
Tobacco users, n (%)	14	(37.8)	6	(24.0)	1.31^b^	0.25
Body Mass Index, M(SD)	22.7	(5.7)	23.8	(3.8)	- 0.89^a^	0.41
Physical activity/week, n (%)						
< 1x	10	(27.0)	7	(28.0)		
1-2x	20	(54.1)	12	(48.0)	0.30^b^	0.86
> 3x	7	(18.9)	6	(24.0)		
**Clinical Information**						
MS course, n (%)						
Relapsing-remitting	26	(70.3)				
Secondary progressive	8	(21.6)				
Clinically isolated syndrome	3	(8.1)				
MS Duration in years, M(SD)						
Relapsing-remitting	6.4	(7.8)				
Secondary progressive	6.4	(5.6)				
EDSS median (range)						
Mild MS	2	0 – 3.5				
Moderate MS	4	4 – 5				
MS treatment, n (%)						
Yes	25	(80.6)				
No	12	(19.4)				

a t-test.

b chi square test.

In order to gain information about walking dynamics with regard to MS-related disability, in a first step, patients were sorted into subgroups displaying moderate and mild disability. Group membership was determined based on the EDSS (Moderate MS Group, EDSS > 3.5, n = 18; Mild MS Group, EDSS < 4, n = 19). Scores below 4 refer to patients who are fully ambulatory, while scores between 4 and 5.5 refer with walking impairment who are able to walk at least 100m without assistive devices [5].

Statistical analyses were performed with SPSS 20.0. Differences in mean walking speed (meters/minute) between MS patients and controls were assessed by a two-way repeated measures ANOVA with the within-subjects factor Test (6MW, 12MW) and the between-subjects factor Group (Mild MS, Moderate MS, Controls). Given a sample size of 60 participants, the use of this model yields a detectable effects size of f = 0.2, when relying on levels of α = 0.05 and β = 0.8. The omnibus test was followed up by two-sided t-tests with Bonferroni-corrected p-values to avoid Type I error inflation.

More importantly, linear and quadratic trends in the walking profiles were examined. In the data obtained in the current study, a quadratic trend reflected the degree to which the walking profile of each group approximated a U-shaped pattern. The linear trend served as an estimate of deceleration. The analyses explored, whether the distance covered during each test minute varied throughout the respective test, and whether putative linear and quadratic trends differed between groups. To this end, a repeated measures ANOVA with the within-subjects factor Minute (6MW: 1-6; 12MW: 1-12) and the between-subjects factor Group (Mild MS, Moderate MS, Controls) was conducted separately for each test.a In an additional analysis, Pearson correlations were used to test for an association between subjective somatic fatigue (WEIMuS-Score) and walking parameters.

## Results

### Mean walking speed

Healthy participants walked significantly faster than both groups of MS patients (see Table 2 for the total distance walked). In particular, the examined groups differed in their average walking speed, as revealed by a highly significant main effect of Group [F(2,59) = 61.65, p < 0.001]. The ANOVA further showed that overall walking speed was affected by walking duration, as indicated by a significant main effect of Test [F(1,59) = 55.18, p < 0.001]. The Group by Test interaction also reached significance [F(2,59) = 3.34, p < 0.05].

**Table 2:** Total distance covered during the 6MW and the 12MW

	**Controls (n = 25)**		**Mild MS (n = 19)**		**Severe MS (n = 18)**	
**Total distance covered in meters**	**M**	**SD**	**M**	**SD**	**M**	**SD**
6MW	681	88	586	73	422	69
12MW	1305	157	1151	148	792	134

Post-hoc comparisons indicated that the main effect of Group was due to significant differences in walking speed between controls and the Moderate MS Group as well as significant differences between controls and the Mild MS group in both tests. The pattern of group differences was consistent across tests, albeit more pronounced in the 6MW (Table 3).

**Table 3:** Mean walking speed, derived linear and quadratic trend components of walking dynamics

	**Controls (n = 25)**		**Mild MS (n = 19)**		**Statistic**		**Severe MS (n = 18)**		**Statistic**	
	**M**	**SD**	**M**	**SD**	**T**	**p**	**M**	**SD**	**T**	**p**
**6MW**										
Mean speed m/min	113.44	14.59	97.71	12.20	3.80	0.000***	70.41	11.49	10.40	0.000***
Linear trend	-0.42	0.55	-0.37	0.35	-0.33	0.745	-1.00	0.78	2.87	0.006**
Quadratic trend	0.52	0.22	0.32	0.23	2.90	0.006**	0.19	0.26	4.50	0.000***
**12MW**										
Mean speed m/min	108.75	13.10	12.37	12.37	3.31	0.002**	66.01	11.16	11.21	0.000***
Linear trend	-0.00	0.25	-0.22	0.33	2.52	0.016*	-0.73	0.51	5.63	0.000***
Quadratic trend	0.05	0.04	0.05	0.03	0.02	0.983	0.03	0.03	1.68	0.100

Comparisons performed between groups of Mild MS patients and healthy controls, as well as Severe MS Patients and healthy controls.

*p-value < 0.05 ** p-value < 0.01 *** p-value < 0.001.

### Linear trend component: decline in walking speed

**Figure 1:**
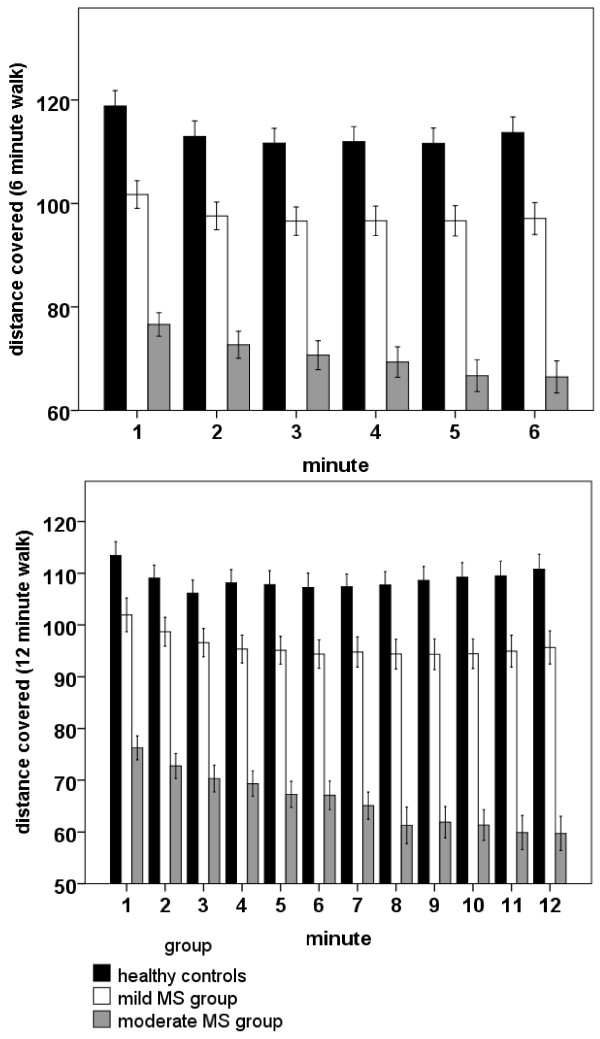
**Velocity profiles of members of the Mild MS, Moderate MS and healthy control group on the 6 min Walking Test (6MW, top) and the 12 min Walking Test (12MW, bottom).** Distance covered is displayed in meters.

In the 6MW, patients continuously slowed down throughout the test, and this decline in speed was significantly pronounced relative to healthy controls (Figure 1). In particular, the duration of walking affected the walking speed, as revealed by a significant main effect of Minute [F(5,295) = 57.76, p < 0.001], with a highly significant linear trend [F(1,59) = 64.26, p < 0.001]. The linear trend, however, was differentially expressed across groups, as revealed by a highly significant Minute by Group interaction [F(2,59) = 7.00, p < 0.01]. Post hoc comparisons indicated a significantly pronounced decline of walking speed in the Moderate MS Group, relative to controls whereas the difference between controls and the Mild MS Group was not significant (Table 3).

The same pattern of a stronger decline in walking speed in patients, relative to controls, was observed in case of the 12MW (Figure 1). In this case, analyses revealed a significant main effect of Minute [F(11,649) = 35.13, p < 0.001], with a significant linear trend [F(1,59) = 46.08, p < 0.001], which was differentially expressed across groups [F(2,59) = 21.54, p < 0.001]. For both groups of patients, the decline in speed was significantly pronounced, relative to healthy controls (Table 3).

### Quadratic trend component: degree of observed U-shape

Overall, participants' performance approximated a U-shaped velocity profile in the 6MW. However, the U-shape was significantly attenuated in MS patients, relative to healthy controls. In particular, there was a highly significant quadratic trend [F(1,59) = 129.73, p < 0.001] which was differentially expressed across groups, as indicated by a highly significant Minute by Group interaction [F(2,59) = 10.82, p < 0.001]. As displayed in Table 3, further testing showed that the quadratic trend was significantly attenuated in both, Mild MS and Moderate MS groups, relative to healthy participants.

The general pacing profile also approximated a U-shaped pattern in the 12MW. In this case the observed U-shape did not vary across groups, and the overall significant quadratic trend [F(1,59) = 89.82, p < 0.001] was not characterized by a significant Minute by Group interaction [F(2,59) = 1.90, p > 0.05].

### Obtained walking dynamics and subjective somatic fatigue

Within the MS group, the self-report parameter of somatic fatigue [15] showed significant correlations with mean walking speed [6MW: r = -0.38; 12MW: r = -0.36, p < 0.05, respectively] and highly significant correlations with the linear decline in walking speed [6MW: r = -0.63; 12MW: r = -0.56, p < 0.001, respectively] in both tests.

In order to verify, whether the association between the linear decline and somatic fatigue was significantly stronger than the association between mean walking speed and somatic fatigue, Steiger's Z-test was used. This revealed that self-reported somatic fatigue was more strongly associated with the decline in speed than with mean walking speed in the 6MW [6MW: Z = 1.98, p < 0.05]. In the 12MW, the difference between the strength of correlations of self-reported somatic fatigue and walking behavior (mean walking speed, linear decline in walking speed) did not reach statistical significance [12MW: Z = 1.50, p > 0.05; two-tailed]. For the control group no significant correlations were obtained (all p-values > 0.05). An exploratory analysis further revealed that there were no significant correlations between the self-report parameters and observed quadratic trends (all p-values > 0.05).

## Discussion

Walking tests are frequently used to examine ambulation in MS patients [1,2]. However, analytic procedures applied to data derived from walking tests, with few exceptions [8-10], remained relatively superficial, leaving temporal walking dynamics almost unexplored. We examined three characteristics of walking behavior in two walking tests, comparing MS patients of mild and moderate disability to healthy controls. Besides the common parameter of mean walking speed, velocity profiles included the linear and the quadratic trend of walking speed during a 6 min Walk (6MW) and a 12 Minute Walk (12MW). The linear trend reflected a measure of deceleration over time, while the quadratic trend estimated the degree to which the walking profile of each group approximated a U-shape. With this analysis, we intended to confirm and extend observations made by Goldman et al. [10] who reported distinct patterns in the walking behavior of MS patients and healthy controls during the 6MW.

### Mean walking speed

Firstly, our results are consistent with findings of Goldman et al. [10], confirming that MS patients walked slower than controls in both tests. In our sample, both, mildly and moderately disabled MS patients displayed attenuated walking speed, relative to controls. As MS commonly affects ambulation, this observation is in line with the extant literature [4,6,9]. The fact that even MS patients with mild disability (Mild MS group) differed significantly from controls in their mean walking speed is noteworthy. This finding hints at an early influence of MS on walking ability in the examined sample and supports previous reports [7,10,16-18].

### Linear trend in walking speed: mean decline

More importantly, results of the current study indicate that differences in walking behavior between MS patients and controls do not only manifest in mean walking speed, but also in altered dynamic walking parameters.

MS patients with moderate disability slowed down more rapidly than controls on both tests. Complementary, MS patients with mild disability displayed a pronounced deceleration in case of the 12MW. These novel findings indicate that continuous deceleration, as reflected by the linear trend in walking speed, represents an additional, clinically relevant feature of impaired ambulation in MS patients. While for patients with moderate disability, the 6MW represents a test which is sufficient to detect this clinical feature, for patients with mild disability, a considerably longer walking duration, as in the 12MW, seems necessary.

The linear decline in walking speed was significantly pronounced in patients, relative to healthy controls, which indicates that deceleration represents a clinically relevant feature. However, the current study provides further original information on the utility of this parameter. In particular, the linear decline in walking speed was highly correlated with subjective somatic fatigue. This provides convergent evidence, indicating that the pronounced deceleration also represents a good estimate of patients' subjective constraints. It is especially noteworthy that linear deceleration showed *highly* significant correlations with subjective somatic fatigue (all p-values < 0.001), whereas the commonly used parameter of mean walking speed showed only *minor* associations with somatic fatigue (all p-values < 0.05). Since these correlations differed significantly from each other in the 6MW^b^, linear deceleration may in fact represent a more suitable parameter to assess somatic fatigue than mean walking speed. This appears plausible, since the dynamic notion of deceleration seems more congruent with the phenomenon of motor fatigue, than the mean walking speed. In sum, our results indicate that deceleration reflects a potentially useful parameter, which is suitable for the assessment of somatic fatigue. As such, our findings provide strong support for the notion of Goldman et al. [10], who suggested to interpret the decline in walking speed as an indicator of fatigue in MS patients.

### Quadratic trend in walking speed: degree of observed u-shape

We also found a U-shaped velocity profile (quadratic trend) across both tests in all groups combined. A U-shaped pacing strategy is a known phenomenon in healthy individuals [11]. As hypothesized, the degree of the U-shape was attenuated in MS patients, relative to controls in the 6MW. Even MS patients with mild disability showed an attenuated U-shape relative to controls on this test. This indicates, that the U-shaped profile can also be regarded as a clinically relevant parameter in MS. However, compared to the parameter of linear deceleration, it does not appear to be as informative, since no association with subjective fatigue was obtained and differences in the U-shaped pattern between the studied groups were not found in the 12MW. Nevertheless, since studies in which this dynamic characteristic is utilized are sparse, the current work provides original results on a new, promising clinical feature, worthy to be explored in more detail in the future.

### Which walking test to use?

Recently, it has been suggested that a brief test including only two minutes of walking (2MW) represents a sufficient measure of walking ability in MS patients [12]. This suggestion was based on a high correlation between the distances covered during the 2MW and the 6MW [12]. Given this correlation, such a suggestion appears feasible when considering the total distance walked. Nevertheless, results of the current study suggest that the choice of which test to use depends on the walking parameter which is supposed to be assessed. For practical reasons, the parameter of mean walking speed, or total distance covered during a given amount of time, has received most attention in research on ambulation in MS. The current study provides novel findings, according to which further parameters can be derived from standard walking tests, which are of high clinical relevance. Particularly the linear trend, as a measure of deceleration, appears to be promising as an estimate of impaired walking ability, since it can be easily derived from a standard walking test. However, since it is not necessarily warranted to derive this measure from a test of only two minutes duration, the current findings do not provide direct support for the suggestion to drastically shorten common tests. In line with this conclusion, Motl et al. [8] have reported that a further putatively relevant parameter, i.e. oxygen consumption, does not reach a steady state within the first two minutes of walking, but remains unaltered only after the third minute. The latter authors suggest that the 2MW functions as a measure of primarily anaerobic and the 6MW as a measure of primarily aerobic performance. Based on our results obtained in case of the 6MW, particularly this test appears to be a feasible measure to capture both, well established parameters, such as the total distance walked, as well as parameters which vary during the walking test. Our findings indicate that an abbreviation of the test duration might result in the loss of potentially important information on dynamic walking behavior, which to date has remained relatively unexplored. Dynamic parameters could serve as a means to increase sensitivity of walking tests to abnormal walking behavior within MS patients.

### Limitations and future directions

In an innovative study Phan-Ba et al. [18] have recently utilized the combination of a timed 500 meter walk (T500MW) and a timed 25 foot walk (T25FW) to assess deceleration in MS. The authors quantified deceleration by computing a *combined* deceleration index, consisting of the ratio of walking speed during the last 100 meters of the T500MW and throughout the T25FW. The authors suggest that such a ratio may be particularly useful to assess ambulation impairment during late stages of MS. Hence, future studies which distinguish between MS subtypes and which explore methodological means to derive further parameters of dynamic walking characteristics may be warranted.

While the current results are in accord with this assumption, they are to be interpreted in the context of few limitations. In particular, it should be noted that the quantification of walking behavior was achieved through visual inspection by an examiner equipped with a stop-watch. This setting was chosen over the one in which a patient is accompanied by an examiner equipped with a precise measurement wheel, to reduce a putatively confounding influence of the setting on walking performance. It is also the approach commonly used in the clinic where the work was carried out. On the other hand, it can be argued that visual inspection may also have an effect on walking performance, and the use of a measurement wheel would have provided a technically more precise measure of walking speed. Hence, the examiner could either have accompanied the patient with the measurement wheel, or the patient could have been equipped with the measurement wheel. Either option may represent a useful alternative to the method implemented in the current study. This fact may be regarded as a caveat to the interpretation of the current results.

Finally, it needs to be noted that the walking parameters explored in the current study imply an alteration of the common nomenclature used in the literature. Performance on walking tests is usually referred to in terms of the total distance covered during a given amount of time. For the use of walking behaviour which changes throughout the test (e.g. deceleration) as a test parameter, a nomenclature involving walking speed appeared more feasible. Nevertheless, when it comes to the interpretation of the current results, it needs to be considered that this nomenclature does not match the one of the extant literature on common walking parameters.

## Conclusions

Our findings highlight the necessity of thoroughly considering the diagnostic goal of a walking test. It is known that the parameter of mean walking speed can be reliably derived from relatively short tests, e.g. the 2MW [12]. However, this parameter might oversimplify the concept of walking impairment, and only tests of longer duration, such as the 6MW, can be used to derive dynamic parameters, which are potentially more informative. As an augmentation to the current standard of measures of walking ability, we contribute a method of quantifying dynamic walking patterns, such as the linear decline in walking speed (linear trend). The linear trend could serve as a surrogate parameter of motor fatigue. It might be better suited than the total distance walked because of its significantly higher correlation with patients' subjective somatic fatigue in the 6MW. Our results support the choice of a longer test duration (12 min) only for MS patients with mild disability (estimated by an EDSS score of less than 4). A shorter test duration (6 min) appears to be sufficient to examine dynamic walking characteristics in MS patients with potentially more moderate deficits (estimated by an EDSS score greater than 3.5). The early registration of changes in walking ability is a key to therapy aimed at minimizing the impairment of mobility. We recommend further research to more thoroughly explore abnormal dynamic walking features in MS, and to establish normative data for these parameters.

## Endnotes

^a^ The linear trend (first order polynomial trend component) describes the mean slope of the velocity profile [8]. An example of the calculation: **D1, D2**, … **Dn** reflect the distance covered during the **first**, **second**, …, **n-th** minute of the walking test. Calculation of the linear trend in the 6MW can be achieved with this formula: LinearTrend^6MW^ = ((-5*D1) + (-3*D2) + (-1*D3) + (1*D4) + (3*D5) + (5*D6))/70 Different formulas were utilized for the 12MW and the quadratic trend [19].

^b^ The fact that the difference between the correlation of linear trend of walking speed and subjective somatic fatigue and the correlation of mean walking speed and subjective somatic fatigue reached statistical significance only in the 6MW but not in the 12MW may be a power issue.

## Competing interests

The authors declare that they have no competing interests. This publication was funded by the German Research Foundation (DFG) and the University of Bayreuth in the funding program Open Access Publishing.

## Author’s contributions

JB conceived of the study, participated in its design, coordination and data acquisition and drafted the manuscript. PK performed the statistical analysis and helped to draft the manuscript. UM participated in the design of the study, reviewed the statistical analysis and revised the manuscript. UH participated in the acquisition of data and revised the manuscript. PO participated in the design of the study. OH participated in the design of the study, reviewed the statistical analysis and revised the manuscript. All authors read and approved the final manuscript.

## Pre-publication history

The pre-publication history for this paper can be accessed here:

http://www.biomedcentral.com/1471-2377/12/161/prepub

## References

[B1] Kieseier BC Pozzilli C Assessing walking disability in multiple sclerosis Mult Scler 2012 18 914 924 10.1177/1352458512444498 22740603

[B2] Bethoux F Bennett S Evaluating Walking in Patients with Multiple Sclerosis. Which Assessment Tools Are Useful in Clinical Practice? Int J MS Care 2011 13 4 14 10.7224/1537-2073-13.1.4 PMC3882949 24453700

[B3] Wetzel JL Fry DK Pfalzer LA Six-minute walk test for persons with mild or moderate disability from multiple sclerosis: performance and explanatory factors Physiother Can 2011 63 166 180 10.3138/ptc.2009-62 22379256 PMC3076913

[B4] Savci S Inal-Ince D Arikan H Guclu-Gunduz A Cetisli-Korkmaz N Armutlu K Karabudak R Six-minute walk distance as a measure of functional exercise capacity in multiple sclerosis Disabil Rehabil 2005 27 1365 1371 10.1080/09638280500164479 16372431

[B5] Kurtzke JF Rating neurologic impairment in multiple sclerosis: an expanded disability status scale (EDSS) Neurology 1983 33 1444 1452 10.1212/WNL.33.11.1444 6685237

[B6] Chetta A Rampello A Marangio E Merlini S Dazzi F Aiello M Cardiorespiratory response to walk in multiple sclerosis patients Respir Med 2004 98 522 529 10.1016/j.rmed.2003.11.011 15191037

[B7] Motl RW Balantrapu S Pilutti L Dlugonski D Suh Y Sandroff BM Symptomatic correlates of six-minute walk performance in persons with multiple sclerosis Eur J Phys Rehabil Med 2012 in press. 22820825

[B8] Motl RW Suh Y Balantrapu S Sandroff BM Sosnoff JJ Pula J Evidence for the different physiological significance of the 6- and 2-min walk tests in multiple sclerosis BMC Neurol 2012 12 6 10.1186/1471-2377-12-6 22380843 PMC3313866

[B9] Schwid SR Thornton CA Pandya S Manzur KL Sanjak M Petrie MD Quantitative assessment of motor fatigue and strength in MS Neurology 1999 53 743 750 10.1212/WNL.53.4.743 10489035

[B10] Goldman MD Marrie RA Cohen JA Evaluation of the six-minute walk in multiple sclerosis subjects and healthy controls Mult Scler 2008 14 383 390 10.1177/1352458507082607 17942508

[B11] Tucker R Noakes TD The physiological regulation of pacing strategy during exercise: a critical review Br J Sports Med 2009 43 e1 10.1136/bjsm.2009.057562 19224909

[B12] Gijbels D Eijnde BO Feys P Comparison of the 2- and 6-min walk test in multiple sclerosis Mult Scler 2011 17 1269 1272 10.1177/1352458511408475 21642370

[B13] Gijbels D Dalgas U Romberg A de Groot V Bethoux F Vaney C Which walking capacity tests to use in multiple sclerosis? A multicentre study providing the basis for a core set Mult Scler 2011 18 364 371 21952098 10.1177/1352458511420598

[B14] ATS statement guidelines for the six-minute walk test Am J Respir Crit Care Med 2002 166 111 117 12091180 10.1164/ajrccm.166.1.at1102

[B15] Flachenecker P Müller G König H Meissner H Toyka KV Rieckmann P "Fatigue" bei Multipler Sklerose. Entwicklung und Validierung des "Würzburger Erschöpfungsinventars bei MS" Nervenarzt 2006 77 165 174 10.1007/s00115-005-1990-x 16160812

[B16] Benedetti MG Piperno R Simoncini L Bonato P Tonini A Giannini S Gait abnormalities in minimally impaired multiple sclerosis patients Mult Scler 1999 5 363 368 10516781 10.1177/135245859900500510

[B17] Martin CL Phillips BA Kilpatrick TJ Butzkueven H Tubridy N McDonald E Galea MP Gait and balance impairment in early multiple sclerosis in the absence of clinical disability Mult Scler 2006 12 620 628 10.1177/1352458506070658 17086909

[B18] Phan-Ba R Calay P Grodent P Delrue G Lommers E Delvaux V Motor fatigue measurement by distance-induced slow down of walking speed in multiple sclerosis PLoS One 2012 7 e34744 10.1371/journal.pone.0034744 22514661 PMC3326046

[B19] Reinard JC Communication research statistics 2006 Thousand Oaks, Calif: SAGE Publications

